# Three‐year cost utility analysis of mini versus standard slings: A trial based economic evaluation

**DOI:** 10.1002/bco2.303

**Published:** 2023-11-13

**Authors:** Mary Kilonzo, Dwayne Boyers, David Cooper, Tracey Davidson, Kiron Bhal, James N'Dow, Graeme MacLennan, John Norrie, Mohamed Abdel‐Fattah

**Affiliations:** ^1^ Health Economics Research Unit University of Aberdeen Aberdeen UK; ^2^ Health Services Research Unit University of Aberdeen Aberdeen UK; ^3^ Department of Urogynaecology University Hospital of Wales Cardiff Wales UK; ^4^ Academic Urology Unit University of Aberdeen Aberdeen UK; ^5^ The Centre for Healthcare Randomised Trials University of Aberdeen Aberdeen UK; ^6^ Usher Institute Edinburgh Clinical Trials Unit University of Edinburgh Edinburgh UK; ^7^ Institute of Applied Health Sciences University of Aberdeen Aberdeen UK

**Keywords:** cost utility analysis, cost‐effectiveness analysis, QALYs, stress urinary incontinence

## Abstract

**Objective:**

To report on the cost‐effectiveness of adjustable anchored single‐incision mini‐slings (mini‐slings) compared with tension‐free standard mid‐urethral slings (standard slings) in the surgical management of female stress urinary incontinence (SUI).

**Patients and Methods:**

Data on resource use and quality were collected from women aged ≥18 years with predominant SUI undergoing mid‐urethral sling procedures in 21 UK hospitals. Resource use and quality of life (QoL) data were prospectively collected alongside the Single‐Incision Mini‐Slings versus standard synthetic mid‐urethral slings Randomised Control Trial (SIMS RCT), for surgical treatment of SUI in women. A health service provider's (National Health Service [NHS]) perspective with 3‐year follow‐up was adopted to estimate the costs of the intervention and all subsequent resource use. A generic instrument, EuroQol EQ‐5D‐3L, was used to estimate the QoL. Results are reported as incremental costs, quality adjusted life years (QALYs) and incremental cost per QALY.

**Results:**

Base case analysis results show that although mini‐slings cost less, there was no significant difference in costs: mini‐slings versus standard slings: £‐6 [95% CI −228–208] or in QALYs: 0.005 [95% CI −0.068–0.073] over the 3‐year follow‐up. There is substantial uncertainty, with a 56% and 44% probability that mini‐slings and standard slings are the most cost‐effective treatment, respectively, at a £20 000 willingness‐to‐pay threshold value for a QALY.

**Conclusions:**

At 3 years, there is no significant difference between mini‐slings and standard slings in costs and QALYs. There is still some uncertainty over the long‐term complications and failure rates of the devices used in the treatment of SUI; therefore, it is important to establish the long‐term clinical and cost‐effectiveness of these procedures.

## 1 INTRODUCTION

Stress urinary incontinence (SUI) is the most common type of UI in premenopausal women. The Nurses' Health Study of almost 24 000 women aged 54–79 years showed 9.2% of women leaked at least monthly.^1^ Although not life‐threatening, UI affects the physical and psychological wellbeing of women^2^ and is associated with a detrimental impact on women's quality of life (QoL).^3^, ^4^ The direct financial burden of UI to women is substantial. In 2009, the annual costs borne by women in the UK were estimated at £230 million or £290 per woman per year.^5^

Treatments for UI include conservative management (such as lifestyle changes and pelvic floor muscle training). If conservative management fails, women may choose among a variety of surgical interventions. Until recently, synthetic mid‐urethral slings (SMUS/mesh/tape) were the standard surgical treatment for female SUI worldwide. Adjustable anchored single‐incision mini‐slings are relatively newer, utilise less mesh and are designed to reduce perioperative morbidity.

The results of our recently published Single‐Incision Mini‐Slings versus standard mid‐urethral slings for surgical treatment of SUI in women: Randomised Control Trial (SIMS RCT)^6^ showed that SIMS were non‐inferior to SMUS with respect to patient‐reported success up to 36‐month follow‐up. SIMS were more likely to be performed with the patient under local anaesthesia (LA) and were associated with less postoperative pain up to 2 weeks after surgery. At 36 months, the percentage of patients with groin or thigh pain was similar in the two groups. However, more women in the mini‐sling group reported dyspareunia, mesh exposure, or further surgery for urinary incontinence or treatment of adverse events. The aim of this article is to report on the cost‐effectiveness of adjustable anchored SIMS compared with tension‐free SMUS in the surgical management of female SUI.

## 2 METHODS

### 2.1 Study design

The cost‐effectiveness analysis was undertaken alongside the SIMS RCT^7^ to determine the cost‐effectiveness of mini‐slings compared with standard slings from a UK National Health Service (NHS) perspective. Cost‐effectiveness was measured using incremental cost‐effectiveness ratios (ICERs) based on quality adjusted life years (QALYs) derived from responses to the generic EQ‐5D‐3L^8^ and the UI‐specific International Consultation on Incontinence Questionnaire Lower Urinary Tract Symptoms Quality of Life (ICIQ‐LUTSqol)^9^ measures over various time points (baseline, 4 weeks and 3 months post‐operatively, and at 15‐, 24‐ and 36‐month post‐randomisation) during the 36‐month follow‐up period. Costs and QALYs accrued in the 24 and 36 months were discounted by 3.5% per annum in line with National Institute for Health and Care Excellence (NICE) recommendations.^10^ All resources were costed in British Pound Sterling (£), using 2018/2019 prices. The methods and results were reported following the Consolidated Health Economic Evaluation Reporting Standards (CHEERS) recommendations, and the analyses followed the health economics analysis plan.^11^

### 2.2 Participants

Full details of the SIMS RCT methods and results are presented elsewhere.^6^, ^7^ In brief, women aged ≥18 years with predominant SUI symptoms who had failed/declined conservative treatment, completed their families and decided to undergo a MUS procedure were recruited from 21 UK hospitals. Exclusion criteria included anterior or apical prolapse ≥ stage 2; previous SUI surgery; predominant overactive bladder (OAB) symptoms; planned concomitant surgery; previous pelvic irradiation; pregnant/planning pregnancy; and an inability to understand consent in English.

### 2.3 Interventions

Women were randomised (1:1) to receive an adjustable, anchored mini‐sling or a tension‐free standard sling. Two main types of mini‐slings were used: AJUST® (CR Bard, Murray Hill, NJ, USA) and ALTIS® (Coloplast Corp., Minneapolis, Minnesota, USA). Standard slings were either retropubic tapes (RP‐TVT) or transobturator tapes (TO‐TVT, inside‐out or outside‐in). Mini‐sling procedures were under LA unless participants requested general anaesthesia (GA). Cystoscopy was performed regardless of the study arm. LA administration and postoperative voiding assessment had standardised guidance.

### 2.4 Resource use and cost collection

The resource use data and costs for the within‐trial analysis included intervention delivery, follow‐up consultations with primary and secondary health care professionals and procedures for subsequent treatment related to treating UI symptoms.

Intervention resource use was collected at the index surgery using case report forms (CRFs) and included type of procedure performed (RP‐TVT/TO‐TVT/mini‐slings), number and type of intervention devices used, staff involved in the surgery (including surgeons, anaesthetists and any supervision provided), time in surgery, type of anaesthesia used (general/spinal/local with intravenous [IV] sedation/LA with oral sedation/LA only), medications, hospital length of stay, details of any catheterisation during and/or after the procedure and any requirements for return to theatre up to the point of discharge. Based on personal communication with trial clinicians, it was established that there were usually three nurses present in the theatre at the surgery (two band five and one band four).^12^

Primary and secondary care resource use post‐discharge was collected using patient postal questionnaires. Primary care resource use included general practitioner (GP) services and referral to other NHS services for subsequent additional specialist management like physiotherapy and district nurses. Secondary care resource use included inpatient re‐admissions related to UI, length of stay, further continence procedures carried out, hospitalisation for adverse events (such as partial or complete tape removal) and outpatient attendances. Where women answered ‘no’ to seeing a health professional, resource use was assumed to be zero. Secondary resource use was also recorded using CRFs completed by research nurses at the point of contact with the hospital. The trial office cross‐checked any details of hospitalisations reported by participants against CRF forms. Any additional hospital contacts reported on questionnaires that were deemed related to UI and validated by the sites were included in the analysis.

### 2.5 Valuing NHS resource use (NHS unit costs)

The base case analysis used a component costing approach for intervention costing. Several surgical devices from different manufacturers were used in the study if they met the pre‐specified criteria in the study protocol.^7^ The unit cost of the devices was based on the list price that the sites purchased them for. Unit costs/prices for all the other resource uses were obtained from the following published sources: British National Formulary^13^ (medications), NHS reference costs^14^ (secondary care resource use post‐discharge), Information Services Division^15^ (theatre costs), Personal Social Services Research Unit Costs of Health and Social Care^12^ (primary care resource use and staff time for intervention costing) and NHS England and Wales (catheters),^16^ as indicated in Table S1. Estimates of resource utilisation were multiplied by unit costs and summed across categories to derive total costs per patient participant.

### 2.6 Measurement of benefits

QALYs were the primary benefit measure for the economic evaluation to enable a cost‐utility analysis (CUA).^17^ QALYs are a composite measure of length and QoL. CUA health‐related QoL was measured using generic (EQ‐5D‐3L) and disease‐specific (ICIQ‐LUTSqol) tools. EQ‐5D‐3L data were collected at baseline, 4 weeks and 3 months post‐intervention, and at 15‐, 24‐ and 36‐month post‐randomisation. EQ‐5D‐3L responses at each timepoint were translated into utility values for QALY calculation using UK general population valuation tariffs.^18^ Disease‐specific QALYs were calculated using responses to the condition‐specific tool ICIQ‐LUTSqol for sensitivity analysis. These data were collected at baseline, 3, 15, 24 and 36 months. ICIQ‐LUTSqol responses were converted into a utility index using a published algorithm.^19^

Both generic and disease‐specific QALYs were calculated as the area under the curve defined by the utility values at baseline and each follow‐up time point,^20^ with linear interpolation between time points. As women did not receive surgery immediately following randomisation, it was assumed that utility remained constant between the point of randomisation and surgery (i.e., women could not generate QALY gains before they received surgery). For women who did not receive surgery, only 15‐, 24‐ and 36‐month post‐randomisation questionnaires were available for QALY calculation.

### 2.7 Data analysis

All components of resource use and costs were described with the appropriate descriptive statistics where relevant: mean and standard deviation (SD) for continuous and count outcomes, n (%) for categorical data. Investigations were carried out for skewed cost data (i.e., a small proportion of participants incurring very high costs), and appropriate distributional assumptions were tested using generalised linear models (GLMs). Two methods were performed to identify the most appropriate distributional family: (1) a modified Park test and (2) the Akaike information criterion (AIC). For costs, the post‐prediction test statistics indicated that the most appropriate family was inverse Gaussian. The goodness of fit statistics based on multiple tests (Pregibon link, Modified Hosmer and Lemeshow and Pearson's correlation) indicated that the log was the appropriate link. The QALY data were analysed using a Gauss family and an identity link. Models were adjusted using a fixed effect for the following minimisation covariates: Previous supervised Pelvic Floor Muscle Training within the last 2 years [PFMT: Yes/No]; age; and baseline QOL data (EQ‐5D‐3L or ICIQ‐LUTSqol as appropriate). The analyses used robust standard errors, clustered by centre. All analyses were conducted using Stata® version 14.1 software (StataCorp LP, College Station, TX, USA).

### 2.8 Missing data

Missing data are a frequent problem in economic evaluations undertaken within a RCT setting, driven by the requirement to use multiple resource use items to calculate costs and the repeated measures nature of questionnaires required to derive total costs and the QALY area under the curve. The mechanism of data missingness was investigated using logistical regression analysis, where the dependent binary variable (missing or not) was regressed on age, minimisation variables, baseline EQ‐5D‐3L utility score and baseline type of UI. Multiple imputations were conducted because more than 5% of both cost and QALY data were missing. Imputations were generated using predictive mean matching drawn from the five k‐nearest‐neighbours (knn = 5); predictive mean matching preserves the distribution of the data and is more robust to violations of the normality assumption. The multiple imputation was run 20 times, generating 20 complete data sets to ensure that stable results and the datasets were combined using Rubin's rules to generate estimates of costs.^21^

### 2.9 Incremental cost per QALY

Results of the CUA are reported as ICERs, given as the difference in costs divided by the difference in QALYs (mini‐slings vs. standard slings). The ICER is assessed against the NICE recommended threshold willingness to pay (WTP) for a QALY of £20 000 per QALY. The joint density of incremental costs and incremental QALYs was derived using non‐parametric bootstrapping. A cost‐effectiveness acceptability curve (CEAC) was used to display the inherent uncertainty surrounding cost‐effectiveness at various WTP threshold values for society's WTP for a QALY.

### 2.10 Sensitivity analyses

Sensitivity analyses were performed to gauge the impact of varying assumptions made in the base case analysis. They included: (1) Complete case analysis was performed to investigate the importance of performing multiple imputations for missing data. (2) QALYs were estimated without adjusting for the wait before the initial surgery. (3) A condition‐specific QoL measure, ICIQ‐LUTSqol, was used to estimate QoL. (4) The discount rate used for costs and QALYs was varied between 0% and 6% in accordance with NICE best practice recommendations. (5) Relaxing the assumption that women who did not have surgery and did not have any CRF completed for follow‐up visits had zero costs.

## 3 RESULTS

### 3.1 Costs

Mini‐sling intervention delivery costs were significantly less than standard slings in terms of the type of anaesthesia administered, the availability of an anaesthetist in the index intervention and recovery time (Table 1). The mini‐slings intervention cost £‐180 [95% CI −£287, −£73] less than standard slings. The follow‐up costs at 3, 15, 24 and 36 months were not significantly different between the groups, with wide confidence intervals and substantial uncertainty. Overall, combining intervention and 36‐month follow‐up costs, the mean cost savings for mini‐slings were ‐£238 per person [95% CI −£508, £32]. It should be noted that the cost data reported in Table 1 are complete cases only, with a substantial volume of missing data. Therefore, the base case cost‐effectiveness analysis considers multiple imputations of missing data.

**TABLE 1:** Average cost complete cases.

Resource	Mini slings mean (SD) *N*	Standard slings mean (SD) *N*	Mini versus standard mean difference^a^ [95% CI]
Total intervention costs (staff time and consumables)	£1069 (410) 298	£1204 (530) 298	£‐180 [−287–−73]
Total 3‐month costs	£21 (45) 231	£28 (80) 204	£‐8 [−19–2]
15‐month primary care^b^	£71 (135) 202	£62 (124) 165	£6 [−22–35]
15‐month secondary care^c^	£184 (618) 275	£174 (684) 265	£21 [−80–121]
Total 15 months (primary and secondary care)	£242 (637) 198	£224 (777) 165]	£45 [−117–206]
Total 15‐month costs (intervention, primary and secondary)	£1367 (593) 173	£1587 (856) 146	£‐218 [−369–−68]
24‐month primary care	£68 (179) 186	£57 (156) 160	£19 [−53–90]
24‐month secondary care	£147 (774) 257	£93 (883) 249	£51 [−64–157]
Total 24 months (primary and secondary care)	£227 (885) 184	£92 (262) 157	£150 [−37–338]
36‐month primary care	£76 (280) 169	£51 (131) 150	£26[−24–76]
36‐month secondary care	£71 (328) 244	£78 (408) 245	£‐8 [−74–57]
Total 36 months (primary and secondary care)	£124 (378) 169]	£100 (298) 149	£48 [−73–169]
Total follow‐up period (intervention, primary and secondary costs^d^)	£1583 (1023) 101	£1830 (1210) 83	£‐238 [−508–32]

Abbreviations: CI, confidence interval; *N*, number of participants; SD, standard deviation.

^a^ Mean differences based on adjusted for baseline EQ‐5D‐3L, PFMT (yes/no) and age and clustered by centre.

^b^ Primary care: visits to general practice doctor, nurse physiotherapist and district nurse.

^c^ Secondary care inpatient re‐admissions related to UI, length of stay, further continence procedures carried out, hospitalisation for adverse events (such as partial or complete tape removal) and outpatient attendances.

^d^ Costs in Year 2 and 3 were discounted at a rate of 3.5%. A negative cost value means mini‐slings cost less than standard slings.

### 3.2 Quality of life measures

Table 2 provides descriptive data of complete case utility scores and QALYs generated by combining utilities with duration of follow‐up. The EQ‐5D‐3L utility scores indicate that the QoL at all time points was higher for the mini‐slings group, including at baseline. However, when calculating incremental QALYs adjusting for baseline imbalances in EQ‐5D‐3L utilities, mini‐slings were associated with fewer QALYs gained over the three‐years (MD: −0.089 [95% CI −0.156, −0.023]. There was little difference in ICIQ‐LUTSqol utilities at any of the measurement time points and there was no evidence of a difference in the derived QALYs between the groups. The Euro Qol Visual Analogue Scale (EQ‐VAS) scores were higher in the standard slings group but the differences in the scores were not statistically significant. These results need to be interpreted in the context that they are based on complete case data across all utility measurement time points, of which between 43% and 47% are missing. It is important therefore to consider multiple imputation for the base case cost‐effectiveness analysis.

**TABLE 2:** Quality of life over 36‐month follow‐up.

Time point	Mini slings mean (SD) *N*	Standard sling mean (SD) *N*	Mini versus standard mean difference, [Cl]^a^
EQ‐5D‐3L			
Baseline	0.860 (0.20) [286]	0.834 (0.25) [284]	
4‐week post‐surgery	0.866 (0.17) [239]	0.838 (0.21) [226]	0.016 [−0.013, 0.046]
3‐month post‐surgery	0.878 (0.19) [255]	0.855 (0.25) [226]	0.002 [−0.031, 0.034]
15‐month post‐randomisation	0.848 (0.24) [249]	0.825 (0.30) [219]	−0.010 [−0.053, 0.030]
24‐month post‐randomisation	0.865 (0.24) [232]	0.816 (0.32) [212]	0.015 [−0.025, 0.055]
36‐month post‐randomisation	0.836 (0.26) [217]	0.821 (0.29) [205]	−0.004 (−0.041, 0.033]
QALYS^b^ ^,^ ^c^	2.376 (0.61) [171]	2.387 (0.67) [159]	−0.089 [−0.156, −0.023]
ICIQ‐LUTSqol			
Baseline	0.94 (0.02) [291]	0.94 (0.02) [284]	
3 months	0.98 (0.02) [248]	0.97 (0.02) [225]	0.002 [−0.001, 0.006]
15 months	0.98 (0.02) [247]	0.98 (0.02) [218]	0.001 [−0.003, 0.005]
24 months	0.98 (0.02) [225]	0.97 (0.02) [208]	0.003 [−0.002, 0.008]
36 months	0.98 (0.02) [217]	0.97 (0.02) [201]	0.003 [−0.002, 0.008]
ICIQ‐LUTSqol QALY^b^ ^,^ ^c^	2.71 (0.15) [179]	2.72 (0.13) [164]	0.002[−0.008, 0.011]

Abbreviations: CI, confidence interval; QALYs, quality adjusted life years; SD, standard deviation.

^a^ Means adjusted for baseline EQ‐5D‐3L, had PFMT (yes/no) and age and clustered by centre.

^b^ Year 2 and 3 QALY discounted at 3.5%.

^c^ QALYs are based on all available data across all time points.

### 3.3 Cost‐effectiveness analysis

#### 3.3.1 Base case analysis

**FIGURE 1:**
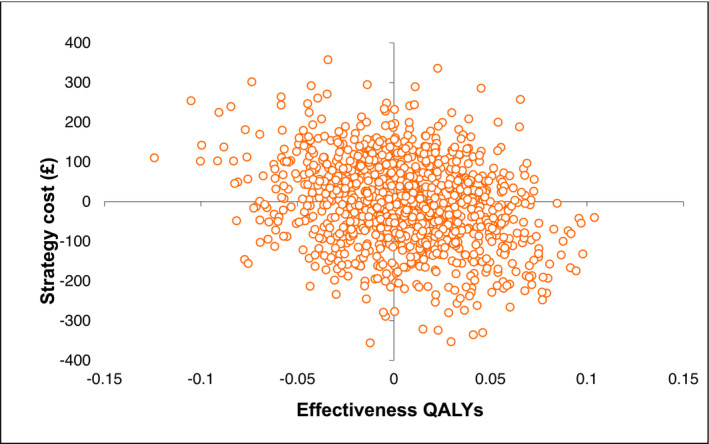
Scatterplot of incremental costs and quality adjusted life years (QALYs) for mini slings compared with standard slings using imputed costs and EQ‐5D‐3L quality of life scores.

The base case cost effectiveness results, based on multiple imputations of missing costs and QALY data, are reported in Table 3. There are no statistically significant differences in costs: mini‐slings versus standard slings: £‐6 [95% CI −228–208] or QALYs 0.005 [95% CI −0.068–0.073] between the groups. The uncertainty in differences in costs and QALYs, and hence cost‐effectiveness, is illustrated by the width of the confidence intervals and in the spread of bootstrapped iterations across all four quadrants of the cost‐effectiveness plane (Figure 1). There is a 56% and 44% probability that mini‐slings and standard slings are the most cost‐effective treatment, respectively, at the £20 000 WTP for a QALY threshold (Figure S1).

**TABLE 3:** Incremental cost‐effectiveness results.

Intervention	Cost	Cost diff (mini vs. standard)^a^ [95% CI]	QALY	QALY diff (mini vs. standard) [95% CI]	ICER (mini vs. standard)	Probability cost effective at society's WTP for QALY threshold		
						£0	£20 000	£30 000
Multiple imputation base case analysis								
Mini slings	£1696		2.347			51%	56%	56%
Standard slings	£1702	‐£6 [−228–208]	2.342	0.005 [−0.068–0.073]	Dominated^b^	49%	44%	44%
Complete case analysis (those with complete cost and QALY data (SIMS *n* = 87 SMUS *n* = 77)								
Mini slings	£1559		2.384			93%	10%	9%
Standard slings	£1769	‐£209 [−493–76]	2.480	−0.096 [−0.227–−0.027]	£2187^c^	7%	90%	91%
Quality of life using ICIQ‐LUTSqol Index imputation sensitivity analysis								
Mini slings	£1696		2.706			51%	48%	49%
Standard slings	£1702	‐£6 [−228–208]	2.708	−0.001 [−0.029–0.023]	£4120	49%	52%	51%
Six percent discount rate								
Mini slings	£1685		2.321			53%	56%	56%
Standard slings	£1693	‐£9 [−227–202]	2.317	0.005 [−0.068–0.071]	Dominated	47%	44%	44%
Unadjusted QALYs								
Mini slings	£1696		2.347			51%	62%	62%
Standard slings	£1702	‐£6 [−228–208]	2.342	0.011 [−0.151–0.013]	Dominated	49%	38%	38%
No assumption of CRF costs for those who did not have surgery								
Mini slings	£1668		2.380			96%	61%	58%
Standard slings	£1757	‐£89 [−192–9]	2.347	0.035 [−0.018–0.082].	Dominated	4%	39%	42%

Abbreviations: CI, confidence interval; Diff, difference; ICER, incremental cost effectiveness ratio, QALY, quality adjusted life year; WTP, willingness to pay.

^a^ Mini slings versus standard slings: Negative cost difference values mean that mini slings costs less while negative QALY difference means mini slings have fewer QALYs.

^b^ Dominated means mini slings costs less and is more effective than standard slings. All analyses are reported on the multiply imputed data set unless otherwise stated.

^c^ The ICER of £2187 suggests that mini slings are not cost‐effective here as the cost saving for a QALY loss is less than the willingness to pay threshold. For the base case analysis there is 56% chance that mini slings would be considered cost effective at £20 000 and £30 000 society's willingness to pay threshold.

#### 3.3.2 Deterministic sensitivity analysis

The results of the sensitivity analysis are included in Table 3. Some of the estimates of incremental cost and QALYs were sensitive to the assumptions made. The results of the complete case sensitivity analysis suggest that on average, mini‐slings cost less than £‐209 [95% CI −493–76] but had fewer QALYs of −0.096 [95% CI −0.227–0.027] than standard slings. The estimates of costs and QALY differences fall mainly in the southwest quadrant of the cost‐effectiveness plane. Mini‐slings had an ICER of £2187 cost savings per QALY loss and 10% chance that they would be considered cost‐effective at the £20 000 WTP threshold to justify a QALY loss.

When the assumption that the secondary care costs of those who did not have surgery were not missing (which was zero) was relaxed, SIMS cost savings increased to £‐89 [95% CI −192–9] and the QALY difference was 0.005 (base–case) to 0.035 [95% CI −0.018–0.082]. The probability that mini‐slings would be considered cost‐effective at the £20 000 WTP threshold was higher than the base case analysis (61% vs. 56%).

The results that used ICIQ‐LUTSqol data indicate that mini‐slings cost less ‐£6 [95% CI −228–208] and were less effective −0.001 [95% CI −0.029–0.023] than standard slings. The ICER was £4120 in cost savings per QALY loss, and the probability that mini‐slings were cost‐effective at the £20 000 WTP threshold was 48%.

The assumptions made in the calculation of the QALY and discount rates applied to the costs did not seem to have an impact on the overall results, with mini‐slings always costing less and having higher QALYs when compared with standard slings. The probability that mini‐slings would be cost effective at the £20 000 WTP threshold ranged between 56% and 62% for these analyses.

### 3.4 Discussion

The base case analysis results indicate that, over the 36‐month follow‐up period, on average, mini‐slings were a less (−£6, 95% CI −£228–£208) costly intervention to deliver because of a combination of shorter surgery and recovery time, less use of GA and lower staff costs. There were no statistically significant cost differences in the use of primary (GP services) or secondary care (hospital visits and further interventions) collected at the 3‐, 15‐, 24‐ and 36‐month follow‐up periods. Therefore, we did not find any statistically significant differences in overall costs (intervention plus follow‐up). There were also no statistically significant differences in QALYs 0.005 (95% CI −0.068–0.073), where the mean effect size of 0.005 QALYs equates to just under an additional 2 days in perfect QoL over 36 months.

There is a high level of uncertainty attached to these results because of the small differences in cost and, more importantly, the small differences in QALYs between the groups. The cost and QALY differences are distributed across all quadrants of the cost‐effectiveness plane. There is a 56% probability that mini‐slings will be considered cost‐effective at the £20 000 and £30 000 WTP threshold values for a QALY, rendering it difficult to draw any firm conclusions regarding 3‐year cost‐effectiveness. Results were also sensitive to decisions about whether to use complete case or multiple imputation analysis and to the choice of QoL measure used (generic or condition‐specific).

The safety of mesh devices has faced significant scrutiny over the last decade, with patients reporting serious adverse events (SAEs) such as tape/mesh exposure, groin/thigh pain and dyspareunia. The mesh scrutiny is primarily regarding its safety profile, with several lawsuits against mesh manufacturers in various countries. In July 2020, Baroness Cumberlege published her much anticipated report, First Do No Harm: Independent Medicines and Medical Devices Safety Review,^22^ looking into the response of England's health‐care system to patients' reports of harm from drugs and medical devices, including transvaginal mesh for surgical treatment of SUI and prolapse. The SIMS trial, including all the follow‐up, was performed during heightened public mesh debate; hence, participants and clinicians are unlikely to have under‐reported AEs. Resource use related to AEs was costed accordingly, and the impact on QoL was captured by the QoL instruments used in the study.

### 3.5 Strengths and limitations

A key strength is that this is the first economic analysis that was conducted on women over 36 months, which captured the costs and benefits related to mini‐slings and standard slings interventions over a longer period. The economic analysis was also undertaken alongside a robust RCT with a multicentre design that included women from all over the United Kingdom, thus increasing the generalisability and validity of the results in the United Kingdom. A comprehensive micro‐costing, which included detailed costing of the intervention device, was undertaken, which adds to the generalisability of the results.

The main limitation was the number of missing costs and QALY data at different time points. Exploratory analysis conducted to predict missingness indicated that data were missing at random, and the base case analysis was conducted using multiple imputation data based on best practice.

### 3.6 Comparison with the literature

Our findings were similar to those reported in a 12‐month cost‐effectiveness analysis: low and non‐significant QALY differences and cost savings for SIMS.^23^ The study reported that mini‐slings had an ICER of £48 419 cost saving per QALY loss with an 80% probability that that mini‐slings would be cost‐effective at the £20 000 WTP threshold. The findings of our study suggest that the mini‐slings follow‐up costs at both 24‐ and 36‐months were higher than standard slings and at 36‐months follow‐up the probability that mini‐slings would be cost effective at 36‐months reduced to 56%.

Another study that used a decision‐analytic model to evaluate the cost‐effectiveness of nine different surgical interventions for the treatment of women with SUI or stress‐predominant MUI concluded that RP‐TVT was less costly and more effective than all other surgical interventions over a lifetime time horizon; therefore, it was a dominant strategy.^24^ The probabilistic results showed that RP‐TVT and traditional slings have the highest probabilities of being cost‐effective across all WTP thresholds over a lifetime time horizon. RP‐TVT remains dominant over a 10‐year time horizon in the cure model. The only major deviation from these findings was when the time horizon was reduced to 1 year. The most cost‐effective surgical intervention was mini‐slings, which was similar to the results that were reported in the 1‐year study.^23^

A brief economic commentary (BEC) reported in a systematic review of single incision operations for urinary incontinence in women identified two studies that reported no difference in clinical outcomes between mini‐slings and TO‐TVT, but the review concluded that mini‐slings may be more cost‐effective than TO‐TVT based on a 1‐year follow‐up.^25^

MUS have been subject to public, political and medical debate over the last 10 years, with scrutiny over the safety of mesh‐based procedures. MUS were suspended in Scotland in 2014 and in the United Kingdom from 2018 to date. The report of the review of the Independent Medicines and Medical Devices (IMMDS) (2020),^26^ led by Baroness Cumberlege, made a number of governance recommendations to be fulfilled prior to the re‐start of MUS in the United Kingdom. Current guidelines^27^ on surgical interventions for urinary incontinence for women considering a surgical procedure for incontinence require that the NICE decision aid on surgery for SUI be used in discussions to promote informed preference and decision‐making. MUS continue to be used in medical practice in the United States and many European countries. There are signals in the literature of late‐onset adverse events and a decline in effectiveness with mesh‐based procedures. Therefore, there is still some uncertainty over the longer time complication and failure rates of the devices used in the treatment of UI, and it is important to establish the long‐term clinical and cost‐effectiveness of SIMS. Ten years' follow‐up of the SIMS study has been funded by Health Technology Assessment (HTA) programme and is underway.

## 4 CONCLUSIONS

The results of this study suggest that the probability that mini‐slings would be cost‐effective is 56% at the £20 000 WTP threshold at 3 years of follow‐up. There is still some uncertainty about the long‐term effectiveness of the devices used to treat SUI in women, and a long‐term study is underway.

## AUTHOR CONTRIBUTIONS

Mohamed Abdel‐Fattah, Mary Kilonzo, John Norrie, James N'Dow and Graeme MacLennan were co‐applicants and contributed to the design of the study. Mohamed Abdel‐Fattah was the chief investigator, Tracey Davidson was the trial manager and David Cooper was the trial statistician. Mary Kilonzo and Dwayne Boyers were responsible for the health economics. Mary Kilonzo conducted the economic analysis and wrote the first draft of this manuscript. Mohamed Abdel‐Fattah, Dwayne Boyers, David Cooper, Tracey Davidson, Kiron Bhal, John Norrie, James N'Dow and Graeme MacLennan all contributed to the drafting of the manuscript.

## CONFLICT OF INTEREST STATEMENT

Dr. Dwayne Boyers reports grants from UK NIHR during the conduct of the study. Ms. Mary Kilonzo reports grants from UK NIHR during the conduct of the study. Kiron Bhal: I have been a speaker and trainer for the following companies in the past Astellas, Pfizer, AMS, Contura, Allergan and others, where I have received honorariums and sponsorship towards attending scientific conferences. Professor James N'Dow reports HTA General Committee 2016–2018. Professor Graeme MacLennan reports grants from UK NIHR during the conduct of the study. Professor John Norrie reports grants from the University of Edinburgh, outside the submitted work; and past and present member of the following: HTA Commissioning Sub‐Board (EOI), NIHR CTU Standing Advisory Committee, NIHR HTA & EME Editorial Board, Pre‐Exposure Prophylaxis Impact Review Panel, EME Strategy Advisory Committee, EME—Funding Committee Members, EME Funding Committee Sub‐Group Remit & Comp Check, HTA General Committee, HTA Funding Committee Policy Group (formerly CSG) and HTA Commissioning Committee. HTA post‐funding committee teleconference 2016–2019; COVID‐19 reviewing 2020. Professor Mohamed Abdel‐Fattah: None in the last 5 years. Before 2015, I have been a speaker, consultant and/or surgical trainer for a number of industrial companies (Astellas, Ethicon, Bard, Pfizer, AMS, Coloplast and others): I have been reimbursed my travel expenses; and on occasions received personal honorariums; proctorship fees and sponsorship towards attending scientific conferences. Research grant from Coloplast managed by the University of Aberdeen. A limited number of my trainees attended pharmaceutical‐sponsored educational/leadership workshops and/or received assistance in presenting their research work at scientific conferences. Was Chairman of the Scottish Pelvic Floor Network (SPFN), which at the time received financial sponsorship from various industrial companies (including all those mentioned above) and non‐profit organisations for its annual meetings and surgical workshops. The SPFN provided an educational grant funding the PI at the highest recruiting site to attend the International Continence Society annual scientific conference in Brazil in 2014. Ongoing: I receive travel sponsorship and occasionally speaker‘s fees from numerous national and international conferences and non‐profit organisations when invited as a guest speaker and/or expert surgeon. In 2019, and at request from NHS Grampian, I attended 2 educational meetings for setting up sacral nerve stimulation service partially funded by Medtronic. I am the Chief Investigator for four NIHR—HTA‐funded studies. I do not hold (and never held) any shares (or similar) in any of the industrial companies (medical or non‐medical). To the best of my knowledge, none of the above have influenced my research or clinical practice.

No other authors declared any potential conflicts of interest.

## Supporting information


**Table S1:** Unit costs.
**Figure S1.** Base‐case analysis cost‐effectiveness acceptability curve:Mini slings versus standard slings.
