# Supplementary material for: Three‐year cost utility analysis of mini versus standard slings: A trial based economic evaluation
Source: BJUI Compass. 2023 Nov 13;5(2):230–9. doi: 10.1002/bco2.303 (PMC10869650; doi:10.1002/bco2.303)
Supplement: Supplementary file 1 — Table S1: Unit costs. Figure S1. Base‐case analysis cost‐effectiveness acceptability curve:Mini slings versus standard slings. [file BCO2-5-230-s001.docx]

**SUPPLEMENTARY MATERIAL**

**Table S1: Unit costs**

| **Resource** | **Unit cost** | **Notes / Source** |
| --- | --- | --- |
| **Intervention resource use** |  |  |
| Mini slings | Several | Price paid by sites for mini sling devices (prices ranged between £350 to £550) |
| Standard slings | Several | Price paid by sites for standard slings devices (Prices ranged between £327 to £584) |
| Consultant | £108 | Cost per working hour based on a 48-hour week (Band 8d)^12^ |
| Associate specialist | £105 | Cost per working hour based on a 48-hour week (Band 8c)^12^ |
| Registrar | £43 | Cost per working hour based on a 48-hour week (Band 6)^14^ |
| Nurse | £37  £28 | Cost per hour (Band 5)^12^  Cost per hour for (Band 4)^12^ |
| General anaesthesia | £22 | Based on cost of drugs and consumables^12^ |
| Spinal anaesthesia | £3 | Based on cost of drugs^13^ |
| Local anaesthesia with sedation | £5 | Based on cost of drugs^13^ |
| Local anaesthesia | £1 | Based on calculation of drugs^13^ |
| Analgesics and anxiolytics | Various prices | Costs based on CRF data provided by participants^13^ |
| Theatre overheads | £420 | Hourly cost excluding supplies^13^ |
| Inpatient stay | £483 | Weighted average cost of elective inpatient excess bed days (LB 51A and B) Vaginal Tape Operations for Urinary Incontinence^14^ |
| Indwelling catheter | £6 | Calculated cost per week of permanent catheter^16^ |
| In and out Catheter | £6 | Unit cost per day of catheter Folysil X-tra (size 14), pack size 1. Assume no additional procedure time required if catheterised during surgery^16^ |
| **Consultations with primary health professionals** | | |
| General Practitioner surgery consultation | £39 | Per surgery consultation lasting  9.22 minutes^12^ |
| General Practitioner telephone call | £24 | Average cost per consultation^12^ |
| Nurse surgery consultation | £9 | Average cost per consultation^12^ |
| Nurse telephone call | £15 | Average cost per consultation^12^ |
| Physiotherapist | £9 | Average cost per consultation^12^ |
| District nurse home visit | £15 | Average cost per consultation^12^ |
| **Consultation with secondary care professionals/procedures for subsequent treatment** | | |
| Outpatient Urology | £108 | Average cost per outpatient attendance: Consultant and Non consultant led. Service code 101 Urology department^14^ |
| Outpatient Gynaecology | £141 | Average cost per outpatient attendance: Service code 502 Gynaecology department^14^ |
| Physiotherapy/Nurse | £58 | Average cost per outpatient attendance: Service code 650 Physiotherapy department^14^ |
| Pain management | £157 | Average cost per outpatient attendance: Service code 191 Pain management department^14^ |
| Neurology | £177 | Average cost per outpatient attendance: Service code 400 Neurology department^14^ |
| Accident and emergency | £168 | Average cost per outpatient attendance: Service code 180 Accident and Emergency department^14^ |
| Indwelling catheter | £6 | Calculated cost per week of permanent catheter^16^ |
| Disposable catheter | £30 | Calculated average cost per week of disposable catheter^16^ |
| Cystoscopy | £1,546 Elective  £1,043 Day case | LB09D Intermediate Endoscopic Ureter Procedures, 19 years and over^14^ |
| Urodynamics | £698 Elective  £368 Day case | LB42A Dynamic Studies of Urinary Tract, 19 years and over^14^ |
| Xray | £31 | DAPF Direct Access Plain Film^14^ |
| MRI | £143 | RD01A Magnetic Resonance Imaging Scan of One Area, without Contrast, 19 years and over^14^ |
| CT Scan | £85 | RD20A Computerised Tomography Scan of One Area, without Contrast, 19 years and over^14^ |
| Follow-up SMUS | £2,904 Elective  £1,549 Day case | Weighted average of LB51 Vaginal Tape Operations for Urinary Incontinence, with CC Score 0 to 2+^14^ |
| Follow-up SIMS | £2,904 Elective  £1,549 Day case | Weighted average of LB51 Vaginal Tape Operations for Urinary Incontinence, with CC Score 0 to 2+^14^ |
| Botox injections | £1,546 Elective  £1,043 Day case | LB14Z Intermediate Endoscopic Bladder Procedures^14^ |
| Tibial Nerve simulation | £2,795 Elective  £1,803 Day case | LB80Z Insertion of Neurostimulator Electrodes for Treatment of Urinary Incontinence^14^ |
| Pubovaginal slings | £4,415 Elective | LB59Z Major, Open or Laparoscopic, Bladder Neck Procedures (Female)^12^ |
| Colposuspension | £4,415 Elective | LB59Z Major, Open or Laparoscopic, Bladder Neck Procedures (Female)^14^ |
| Duloxetine | £0.99 | Cost per tablet^13^ |
| Anti-muscarinic treatment | £0.52 | Average cost per tablet of reported antimuscarinics^13^ |
| Antibiotics | £0.19 | Average cost per tablet of reported antibiotics^13^ |
| Urethral dilation | £1,546 Elective £1,043 Day case | LB14Z Intermediate Endoscopic Bladder Procedures^14^ |
| Insertion of suprapubic catheter | £1,136 Elective  £400 Day case | LB18Z Attention to Suprapubic Bladder Catheter^14^ |
| Urethrolysis | £1,546 Elective  £1,043 Day case | LB14Z Intermediate Endoscopic Bladder Procedures^14^ |
| Complete removal of tape | £3,239 Elective  £1,811 Day case | Weighted average of MA03 Major Open Lower Genital Tract Procedures with CC Score 0-3+^14^ |
| Partial removal of tape | £2,539 Elective  £1,452 Day case | Weighted average of MA04 Intermediate Open Lower Genital Tract Procedures with CC Score 0-3+^14^ |

CC complexity and comorbidity

**Figure S1 Base-case analysis cost-effectiveness acceptability curve:Mini slings versus standard slings**

WTP willingness to pay; QALY quality adjusted life years. At £20,000 society’s WTP for an additional QALY threshold of £20,000 there is a 56% chance that mini slings are cost effective.
